# Osteoclastic effects of mBMMSCs under compressive pressure during orthodontic tooth movement

**DOI:** 10.1186/s13287-021-02220-0

**Published:** 2021-02-25

**Authors:** Jing Wang, Delong Jiao, Xiaofeng Huang, Yuxing Bai

**Affiliations:** 1 Department of Orthodontics, School of Stomatology, Beijing Stomatological Hospital, Capital Medical University, Beijing, 100050 China; 2 Department of Stomatology, Beijing Friendship Hospital, Capital Medical University, Beijing, 100050 China

**Keywords:** Orthodontic tooth movement, Bone marrow mesenchymal stem cells, Osteoclast, Bone remodelling, Compression, Caudal vein injection

## Abstract

**Background:**

During orthodontic tooth movement (OTM), alveolar bone remodelling is closely related to mechanical force. It is unclear whether stem cells can affect osteoclastogenesis to promote OTM. This study aimed to investigate the role of mouse bone marrow mesenchymal stem cells (mBMMSCs) under compression load in OTM.

**Methods:**

A mouse OTM model was established, and GFP-labelled mBMMSCs and normal saline were injected into different groups of mice by tail vein injection. OTM distance was measured using tissue specimens and micro-computed tomography (micro-CT). The locations of mBMMSCs were traced using GFP immunohistochemistry. Haematoxylin-eosin staining, tartrate-resistant acid phosphate (TRAP) staining and immunohistochemistry of Runx2 and lipoprotein lipase were used to assess changes in the periodontal ligament during OTM. mBMMSCs under compression were co-cultured with mouse bone marrow-derived macrophages (mBMMs), and the gene expression levels of Rankl, Mmp-9, TRAP, Ctsk, Alp, Runx2, Ocn and Osterix were determined by RT-PCR.

**Results:**

Ten days after mBMMSCs were injected into the tail vein of mice, the OTM distance increased from 176 (normal saline) to 298.4 μm, as determined by tissue specimen observation, and 174.2 to 302.6 μm, as determined by micro-CT metrological analysis. GFP-labelled mBMMSCs were mostly located on the compressed side of the periodontal ligament. Compared to the saline group, the number of osteoclasts in the alveolar bone increased significantly (*P* < 0.01) on the compressed side in the mBMMSC group. Three days after mBMMSC injection, the number of Runx2-GFP double-positive cells on the tension side was significantly higher than that on the compression side. After applying compressive force on the mBMMSCs in vitro for 2 days, RANKL expression was significantly higher than in the non-compression cells, but expression of Alp, Runx2, Ocn and Osterix was significantly decreased (*P* < 0.05). The numbers of osteoclasts differentiated in response to mBMMs co-cultured with mBMMSCs under pressure load and expression of osteoclast differentiation marker genes (Mmp-9, TRAP and Ctsk) were significantly higher than those in mBMMs stimulated by M-CSF alone (*P* < 0.05).

**Conclusions:**

mBMMSCs are not only recruited to the compressed side of the periodontal ligament but can also promote osteoclastogenesis by expressing Rankl, improving the efficiency of OTM.

## Background

Orthodontic tooth movement (OTM) is a process based on bone remodelling. After teeth are loaded with a certain mechanical force, bone resorption occurs on the pressure side, while new bone is formed on the tension side [1]. This involves a complex process of bone metabolism, which ultimately results in tooth movement. The rate of OTM is mostly dependent upon osteoclast activity and bone resorption, which is typically referred to as the rate-limiting step [2–4]. Bone resorption is directly related to the following processes: bone voidage, remodelling rate, resorption rate and osteoclast replenishment [5]. There is a close relationship between OTM and osteoclast formation on the pressure side, and the direction of osteoclast differentiation is most primarily related to tooth movement.

Mechanical force is of great significance for maintaining bone tissue and structural integrity, as well as promoting bone maturity [6, 7]. It promotes the occurrence of a series of biological behaviours by regulating signal transduction between cells. For example, under mechanical pressure, osteocytes can promote osteoclast formation through secretion of RANKL mediated by autophagy [8]. Mechanical load can also participate in periodontal physiological function by releasing lyases into the extracellular matrix, such as tartrate-resistant acid phosphatase (TRAP), matrix metalloproteinase-9 (Mmp-9) and cathepsin K (Ctsk), to ensure continuous bone resorption activity.

As osteoclast precursor cells, bone marrow-derived macrophages, which arise from haematopoietic cell lines, play a key role in bone remodelling during tooth movement [9]. Mesenchymal stem cells can regulate macrophage polarization by secreting a variety of biological and immunoregulatory factors to complete bone remodelling [10]. Some studies have confirmed that human-derived stem cells promote osteoclast differentiation in macrophage lines [11]. However, this effect is directly related to the category and amount of cytokine stimulation and to whether stem cells are physically treated [12]. It has been reported that periodontal ligament stem cells (PDLSCs) regulate the polarization of M1 macrophages under mechanical stimulation, which may promote bone remodelling and tooth movement [13]. Growth differentiation factor 15 (GDF15), produced by periodontal ligament cells (PDLCs) in response to stress, promote osteoclast differentiation of macrophage lines [14]. However, many mechanisms by which stem cells regulate osteoclasts and tooth movement remain unclear. For example, bone marrow mesenchymal stem cells (BMMSCs) may function through activating dormant and inhibited cells and secreting immunoregulatory factors. The osteoclast differentiation of bone marrow-derived macrophages by homologous mesenchymal stem cells under stress and unstressed conditions needs to be examined together. It is also unclear whether exogenous and homologous mesenchymal stem cells promote differentiation of osteoclast progenitor cells in the periodontal ligament in response to orthodontic mechanical force.

In this study, homologous mBMMSCs were injected into an animal model of OTM in vivo and co-cultured with mouse bone marrow-derived macrophages (mBMMs) in vitro to explore the mechanism by which stem cells regulate the differentiation of osteoclast precursors to promote tooth movement.

## Methods

### Animals

All animals were 8-week-old inbred ICR mice obtained from Hua Fukang Biotechnology Co., Ltd. (Beijing, China). All experiments were conducted according to animal research procedures through the ethics review process (KQYY-201906-003). Mice were divided into two groups of six mice each. Tail vein injection of mBMMSCs served as the experimental group, and saline injection served as the control group.

### Isolation, culturing and identification of mBMMSCs

Three- to 4-week-old ICR mice were sacrificed through cervical dislocation. The hind legs were dissected and gently cleaned from the adherent soft tissues. Cells from femurs and tibias were flushed with α-MEM. After 24 h, we removed the supernatant and replaced it with fresh complete medium [15].

After culturing in mineralization induction solution for 17 days, mBMMSCs were stained with alizarin red staining solution to identify mineralized nodules. After culture in adipogenic induction solution for 20 days, mBMMSCs were stained with oil red O solution to identify lipid droplets. Mesenchymal stem cell markers of mBMMSCs were identified by flow cytometry with positive markers (CD146, CD105) and negative markers (CD45) using the antibodies CD146 (ab33300, Abcam, UK), CD105 (ab53321, Abcam, UK) and CD45 (ab210273, Abcam, UK).

### Osteoclast precursor cells/mBMMs

Bone marrow content flushed from femurs and tibias of mice was cultured in α-MEM containing 10% foetal bovine serum, 30 ng/ml M-CSF and 1% penicillin-streptomycin. After 24 h, the supernatant was extracted and cultured with the above medium for 2 days, and mBMMs were obtained [16].

### OTM model

Eight-week-old ICR mice were anaesthetized with chloral hydrate (40 mg/kg). Nickel titanium tension springs (Tomy, Japan) were used to provide 30 g force to the left maxillary first molar, and the other end was fixed on the incisor using a resin ball (3M, USA) [17, 18]. mBMMSCs were injected into the tail vein on day 0, and orthodontic force was applied on the same day. On the 5th day, mBMMSCs were administered in a second injection [19]. Mice were given soft food after the operation and sacrificed 10 days after the procedure, and the maxillae were dissected and fixed in 4% paraformaldehyde.

### Caudal vein injection of stem cells

Mouse tail veins were dilated using hot water. One-millilitre syringe was used to draw up a 100 μl/10^7^ mBMMSC suspension, which was injected into the caudal vein at an average speed [20].

### Method of measuring tooth movement

Distances of OTM were determined under a stereomicroscope after 10 days of tooth movement according to the midpoint of the distal marginal crest of the maxillary first molar and the midpoint of the mesial marginal crest of the second molar [21]. A micro-computed tomography (Inveon, Siemens, Germany) scanner and RadiAnt DICOM Viewer (64-bit) software were also used for analysis. A section of the centre of the distal buccal root of the maxillary first molar and the mesial buccal root of the second molar was made, and the distance between the most convex points of the crown on the section was measured as the tooth movement distance. Distances were measured by the same researcher 3 times, and researchers who measured the amount of tooth movement were blinded to the treatment groups.

### Histological evaluations

Fixed mouse maxillae were decalcified with 10% EDTA (pH 7.2) for 30 days and embedded in paraffin. Specimens were sliced at 5-μm thickness, followed by green fluorescent protein (GFP, ab1218, Abcam, UK), Runx2 (Runt-related transcription factor, A2851, ABclonal, China) and Lpl (lipoprotein lipase, A16252, ABclonal, China) immunohistochemistry, H&E staining (Beyotime, China) and TRAP staining (Solarbio, China).

In the HE staining, we selected a maximum width of the periodontal ligament from the cementum-enamel junction to the apex adjacent to the crown in the distal buccal root compression side. The width of the periodontal ligament was measured in this area.

In TRAP staining, a region of interest was selected for the number of osteoclasts from the cementum-enamel junction to the apex in the distal buccal root compression side of the first molar.

The region of one-third distance from the cementum-enamel junction to the apex adjacent to the crown was selected to determine the number of positive cells in the proximal buccal root of the first molar in GFP, GFP-Runx2 and GFP-Lpl double immunohistochemical staining for cell counting.

### Transwell co-culture system of mBMMs induced by pressurized mBMMSCs

The next experiment was divided into four groups, detailed as follows: (1) blank control, mBMMs + M-CSF; (2) negative control, mBMMs + mBMMSCs +M-CSF; (3) experimental, mBMMs + mBMMSCs + compression + M-CSF; and (4) positive control, mBMMs + mBMMSCs +RANKL+M-CSF.

In this co-culture system, mBMMs were seeded into the lower Transwell chambers at 3 × 10^4^ cells/cm^2^, induced by M-CSF (50 ng/ml), and mBMMSCs were seeded into the upper Transwell chambers at 3 × 10^4^ cells/cm^2^. mBMMSCs covered with glass coverslips were subjected to 2 g/cm^2^ force in the stem cell with compression group. In the RANKL group, mBMMs were induced by 100 ng/ml RANKL. After 7 days of culture, TRAP staining and PCR detection were performed in all four groups.

### Reverse transcription polymerase chain reaction (RT-PCR)

Total RNA of cells was extracted with TRIzol reagent (Ambion, USA) using the PrimeScript™ RT Reagent Kit (Perfect Real Time) (TaKaRa, Japan) to synthesize DNA (cDNA), with RNA as the template. The cDNA obtained was subjected to PCR using SYBR Green with the following primer sequences shown below (Table 1). All of the above procedures were performed in strict accordance with the manufacturer’s instructions.

**Table 1:** The primer sequences

Gene (mouse)	Sequence (5′ to 3′)
GAPDH	Sense ACCCTAAGGCCAACCGTGAAAAG
	Antisense CATGAGGTAGTCTGTCAGGT
RANKL	Sense GCAGAAGGAACTGCAACACA
	Antisense TGATGGTGAGGTGTGCAAAT
TRAP	Sense TGGTCATTTCTTTGGGGCTTATCT
	Antisense GCTACTTGCGGTTTCACTATGGA
MMP-9	Sense CGTGTCTGGAGATTCGACTTGA
	Antisense TTGGAAACTCACACGCCAGA
CTSK	Sense TGACCACTGCCTTCCAATAC
	Antisense CTCTGTACCCTCTGCATTTAG
Runx2	Sense GAGGCCGCCGCACGACAACCG
	Antisense CTCCGGCCCACAAATCTCAGA
Osterix	Sense ATTCTCCCATTCTCCCTCCCT
	Antisense GGAAGGGTGGGTAGTCATTTGC
ALP	Sense TGCCTACTTGTGTGGCGTGAA
	Antisense TCACCCGAGTGGTAGTCACAATG
OCN	Sense AGCAGCTTGGCCCAGACCTA
	Antisense TAGCGCCGGAGTCTGTTCACTAC

### Statistical analysis

All data were analysed using SPSS 22.0 software. Independent-sample *t* test or one-way analysis of variance (ANOVA) with Bonferroni correction was used to analyse significant differences with a normal distribution. Wilcoxon-Mann-Whitney tests were performed to determine statistical significance in data that was not normally distributed. GraphPad Prism 8 was used for analysis, and *P* < 0.05 indicated statistical significance.

## Results

### Characterization of mBMMSCs

**Fig. 1:**
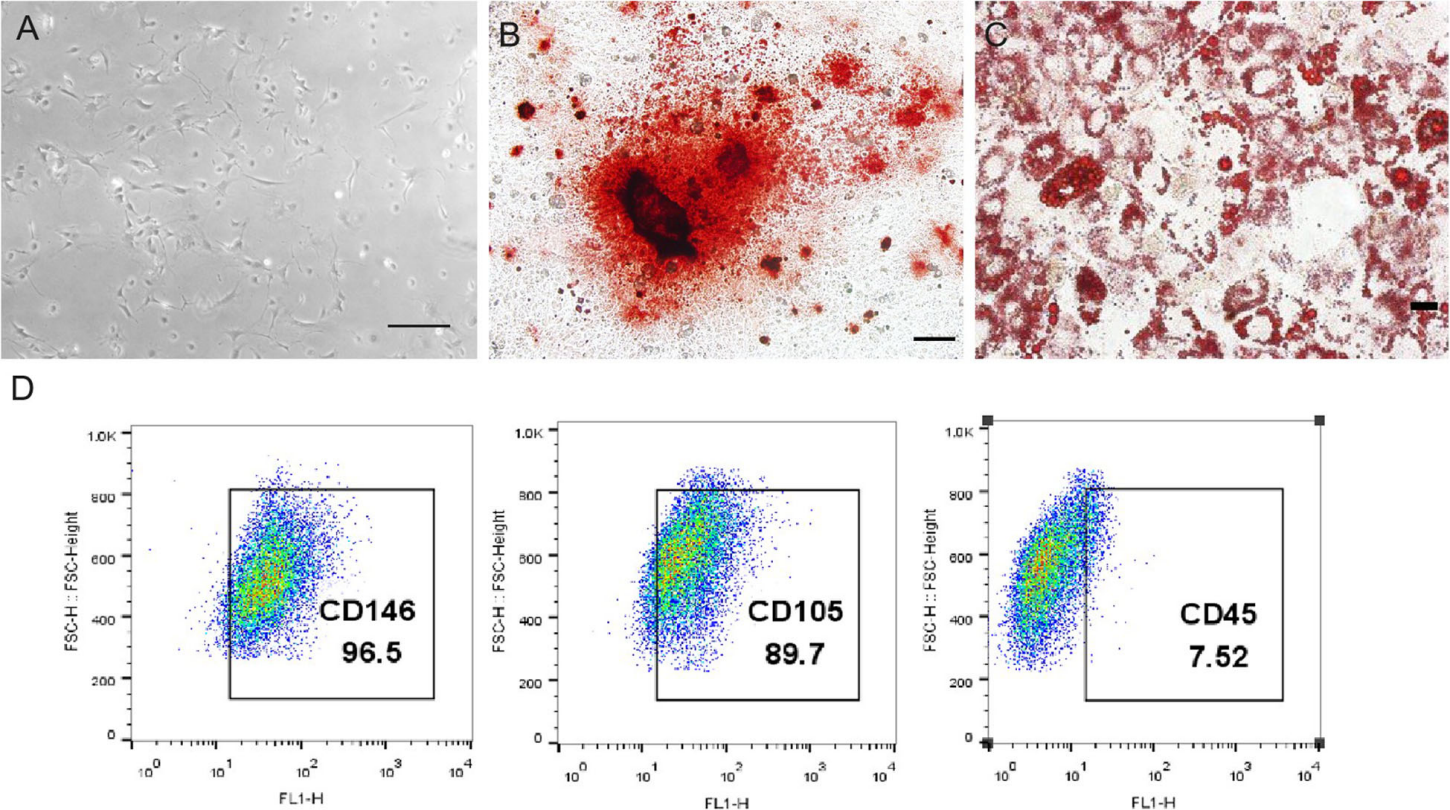
Characterization of mBMMSCs. **a** The first generation of mBMMSCs cultured in vitro. **b** Alizarin red staining indicated the osteogenic differentiation potential of mBMMSCs, showing calcified nodules formed 14 days after osteogenic induction. **c** Lipid droplets formed by mBMMSCs after adipogenic induction for 20 days (oil red O staining). **d** Flow cytometry indicated that mBMMSCs proliferated in vitro and highly expressed mesenchymal stem cell surface markers (CD146 and CD105) but did not express the haematopoietic stem cell surface marker CD45. The scale bars in **a** and **b** are 200 μm, and the scale bar in **c** is 50 μm

mBMMSCs isolated from the mouse bone marrow cavity exhibited multipotential differentiation characteristics (Fig. 1a). After 14 days of osteogenic induction, calcified nodules (Fig. 1b) were identified by alizarin red S staining. After 20 days of adipogenesis induction, lipid droplets (Fig. 1c) were identified by oil red O staining. Flow cytometry revealed that mBMMSCs highly expressed mesenchymal stem cell surface markers (CD146 and CD105) but did not express the haematopoietic stem cell surface marker CD45 (Fig. 1d).

### Injection of homologous stem cells in vivo accelerates OTM in ICR mice

**Fig. 2:**
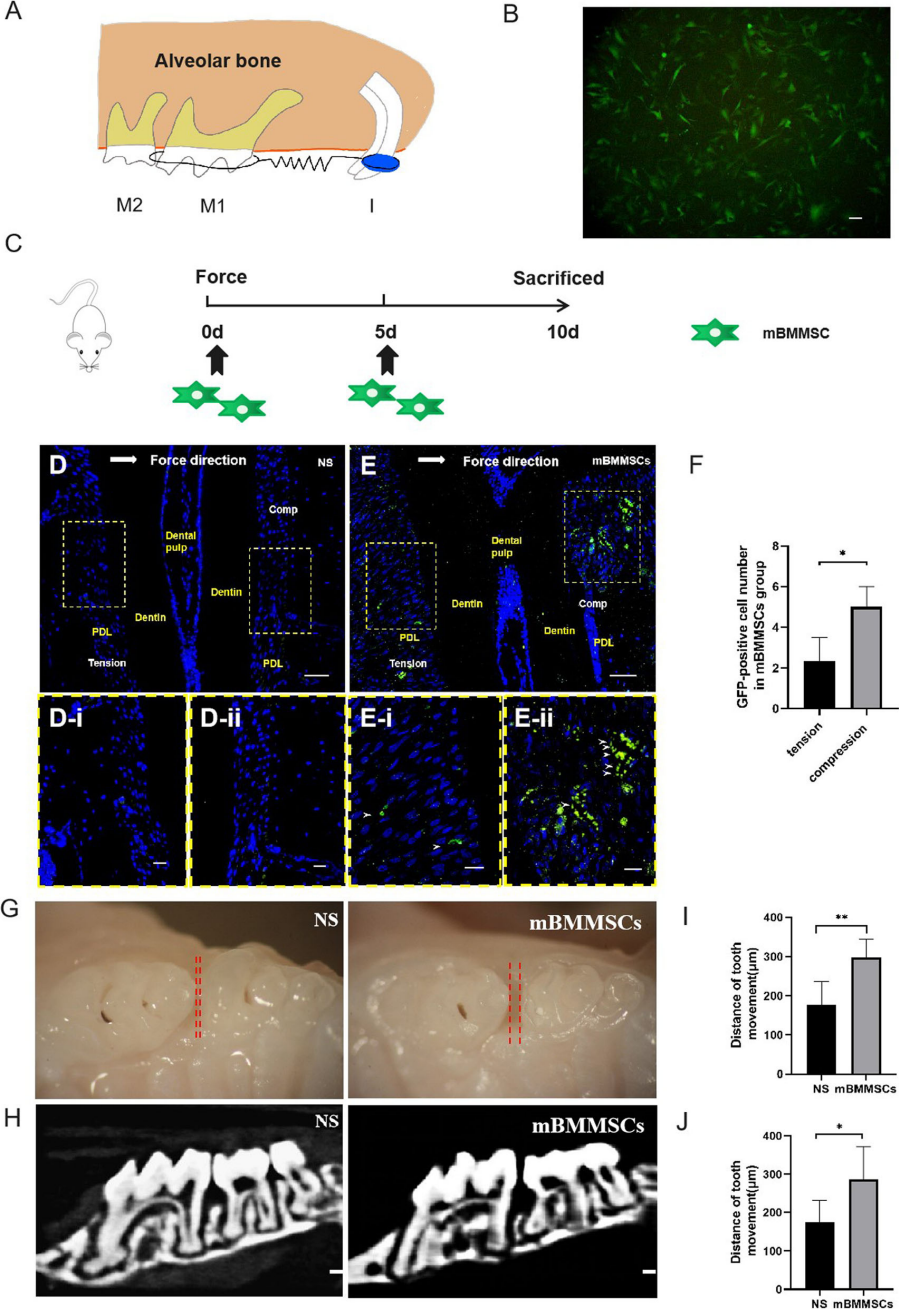
Mice that received intravenous infusion of mBMMSCs exhibit increased OTM. **a** OTM model in mouse oral cavity. The mouse maxillary incisor was used as anchor, 30 g force was applied, and the left maxillary first molar was moved mesially with a spiral spring. M1, first molar; M2, second molar; I, Incisor. **b** Fluorescence microscopy images showed that GFP was expressed in mBMMSCs transfected with GFP-lentivirus before injection. **c** The experimental schedule of OTM. Two groups of mice received an orthodontic stress device for 10 days. mBMMSCs were infused into the tail vein on days 0 and 5 and then sacrificed 10 days from day 0. **d**, **e** After applying orthodontic force for 3 days, immunohistochemistry revealed green fluorescence-positive cells on the tension and compression side periodontal ligament of orthodontic teeth in the saline injection and GFP-labelled mBMMSC groups. The large white arrow represents the direction of orthodontic force, the yellow dotted frame indicates the enlarged area, and the small white arrow represents GFP-positive mBMMSCs. d-i, e-i The tension side of the periodontal ligament, and d-ii, e-ii the compression side of the periodontal ligament, and blue indicates the nucleus, as shown by DAPI staining. Comp, compression surface; Tension, tension surface; PDL, periodontal ligament; NS, injection normal saline group; mBMMSCs, injection stem cell group. *N* = 5 mice/group. **f** Statistical results of green fluorescence-labelled mBMMSCs in the periodontal ligament of the compression side and tension side of the first molar in the mBMMSC-injected group. *P < 0.05 versus the tension side. **g** Occlusal view of the molar area 10 days after OTM. The first molars were moved mesially far away from the second and third molars (*N* = 5 mice/group). **h** Micro-CT analysis demonstrated movement of the first molar in the OTM of both groups. **I**. **j** Statistical results of OTM of mice in the normal saline and stem cell injection groups. NS, injection normal saline group; mBMMSCs, injection stem cell group. The red dotted line represents the tooth movement distance. *P < 0.05 versus NS group, **P < 0.01 versus NS group. Scale bars in **b**, **d** and **e** are 50 μm, the scale bars in d-i, d-ii, e-i and e-ii are 20 μm, and scale bars in **h** are 500 μm

To observe the effect of stem cells on OTM, we established a mouse OTM model [18] (Fig. 2a). Experimental mice were injected with homologous mBMMSCs, and orthodontic force was applied on the same day. mBMMSCs were administered again on day 5, and mice were sacrificed on day 10 (Fig. 2c).

To trace mBMMSCs, a lentivirus carrying GFP was transfected into mBMMSCs in vitro before injection. After 12 h, most mBMMSCs were green under a fluorescence microscope (Fig. 2b). Three days after injection under orthodontic force, immunohistochemical results showed that green fluorescent protein was expressed in the periodontal ligament of orthodontic roots, and the number of mBMMSCs with green fluorescence on the compressed side was greater than on the tension side (Fig. 2e, f), while no obvious GFP-labelled-mBMMSC expression was detected in the orthodontic periodontal ligament in the saline injection group (Fig. 2d), indicating that transplanted mBMMSCs were primarily recruited to the pressure side periodontal ligament and might play a role there (Fig. 2e). In the mBMMSC injection group, the average number of GFP-labelled mBMMSCs on the tension side was 2.2/area, while the pressure side exhibited significantly increased numbers (5/area) (*P* < 0.05, Fig. 2f).

In tissue specimens, the average tooth movement distance of the left first molar in the stem cell injection group was 298.4 μm, while the distance in the saline injection group was much lower (176 μm) (Fig. 2g, i). In addition, we applied micro-CT analysis to further verify this observed difference. The orthodontic tooth movement distance in the stem cell injection group (302.6 μm) was greater than in the normal saline group (174.2 μm, Fig. 2h, j). There was a significant difference between the two groups as determined by histology and micro-CT (*P* < 0.01, Fig. 2i, j).

### mBMMSCs enhance osteoclast differentiation on the pressurized side of orthodontic teeth

**Fig. 3:**
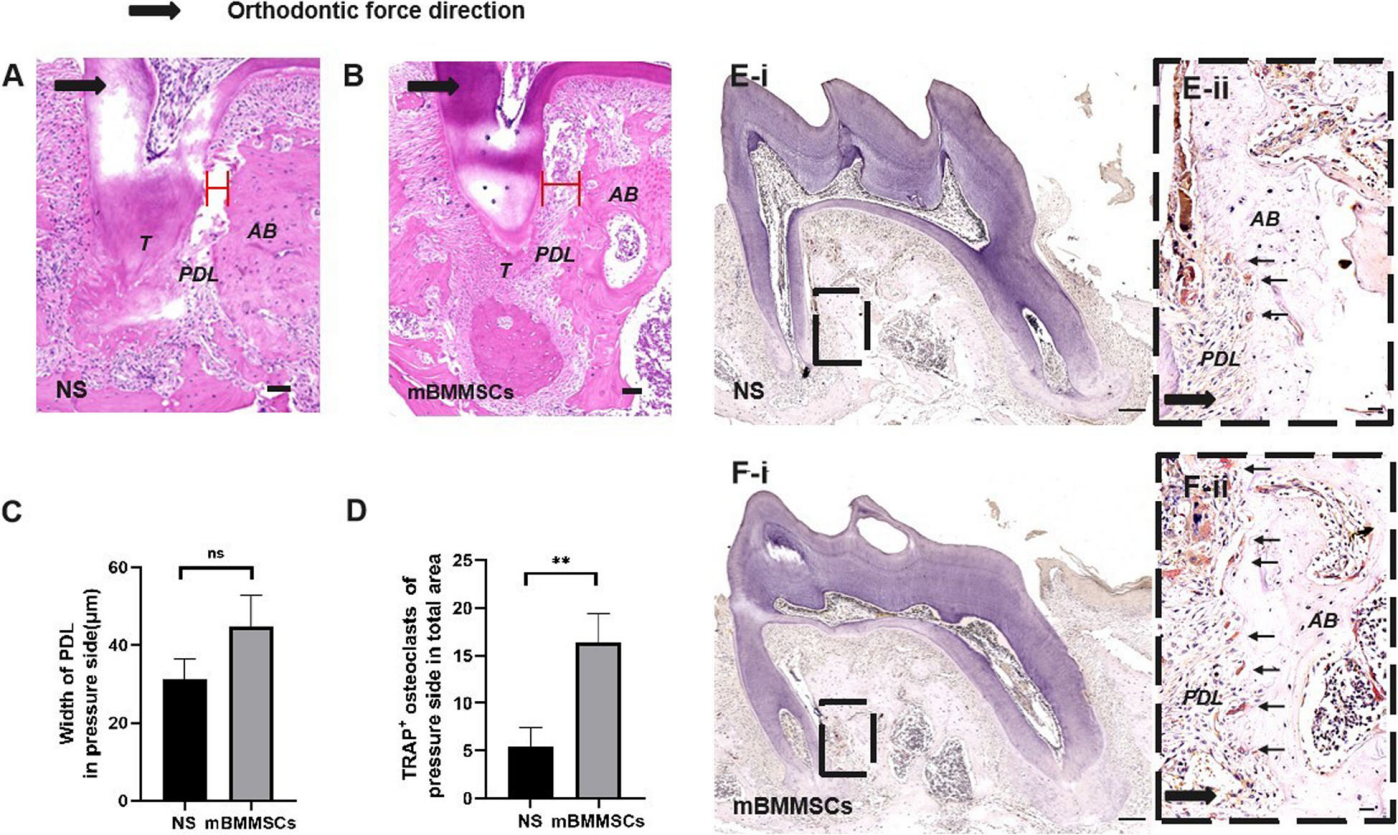
mBMMSCs increase the number of osteoclasts in mice after 10 days. **a**–**c** The width of the periodontal ligament on the pressure side of the distal buccal root of the first molar in the mBMMSC injection group was larger than in the saline injection group. The large black arrows represent the direction of orthodontic force. AB, alveolar bone; T, tooth; PDL, periodontal ligament (*N* = 5/group). **d** Statistical results of osteoclasts in tartrate-resistant acid phosphatase (TRAP) staining images of two groups of pathological sections. The number of osteoclasts produced along the pressure site of the mBMMSC group was much greater than in the saline injection group. **P < 0.01 versus NS group. **e**, **f** The number of TRAP-positive osteoclasts was increased on the pressure side in the stem cell injection group after 10 days. The large black dotted frame shows a high-magnification view of the small box area. The large black arrows represent the direction of orthodontic force, and the small black arrows represent TRAP-positive multinucleated osteoclasts (*N* = 5/group). AB, alveolar bone; PDL, periodontal ligament. The scale bars in **a**, **b**, **e-i** and **f-i** are 50 μm, and the scale bars in **e-ii** and **e-ii** are 20 μm

The same orthodontic force was applied on the first molars in the both groups of mice for 10 days. H&E-stained sections showed that the average width of the periodontal ligament on the compressed side of the distal root of the mBMMSC group was larger than the normal saline group. There was no significant difference between the two groups, but there was a trend towards increased periodontal ligament (PDL) in the mBMMSC group (Fig. 3a–c). The number of osteoclasts produced on the distal root pressure side of the stem cell injection group (16.4 cells/area) were higher than in the saline injection group (5.6 cells/area) (*P* < 0.01) (Fig. 3d–f), indicating that stem cells promote osteoclast differentiation under compression.

### GFP colocalizes with transplanted mBMMSCs expressing Runx2 and Lpl

**Fig. 4:**
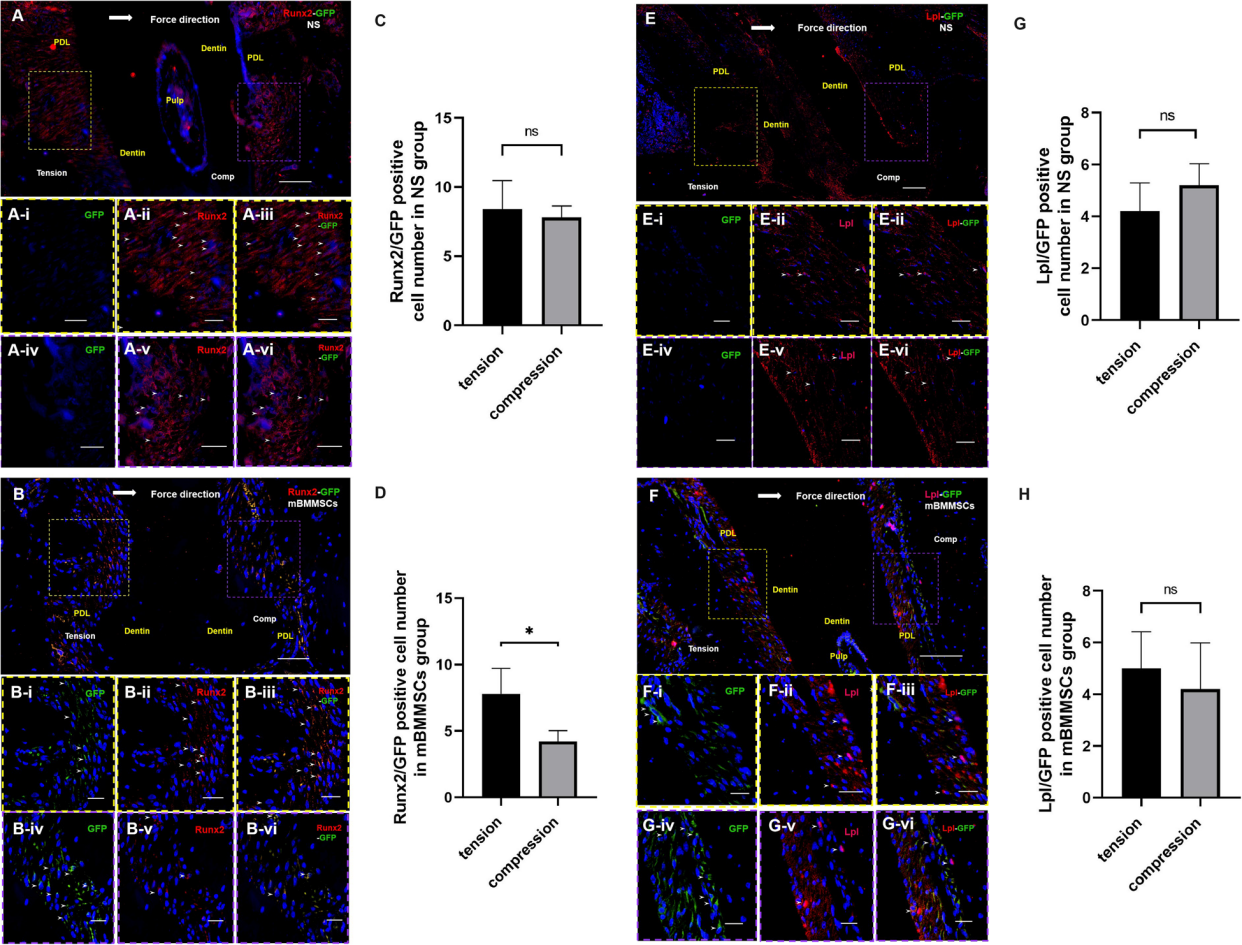
The status of mBMMSCs after colocalization of GFP, Runx2 and Lpl. **a**, **b** After the infusion of normal saline and stem cells, on the third day after orthodontic application, Runx2-GFP-positive cells were expressed on the compression and tension sides of the distal roots of the first molars of mice. **c**, **d** Statistical results of Runx2-GFP-labelled mBMMSCs in the periodontal ligament of the compression side and tension side of the first molar in the mBMMSC and normal saline injection groups. *P < 0.05 versus the tension side. **e**, **f** After normal saline and stem cell infusion, expression of Lpl-GFP-positive cells on the compression and tension sides of the distal root of the first molar of the mouse was observed on the third day after orthodontic application. **g**, **h** Statistical results of Lpl-GFP-labelled mBMMSCs in the periodontal ligament of the compression side and tension side of the first molar in the mBMMSC and normal saline injection groups. **a**, **b**, **e**, **f** The yellow dashed box indicates the magnified area of the tension side, and the purple dashed box indicates the magnified area of the compression side. The large white arrow represents the direction of orthodontic force, and the small white arrow shows positive cells (*N* = 5/group). **a-i**, **b-i**, **e-i**, **f-i** DAPI staining colocalized with GFP staining; **a-ii**, **b-ii** DAPI staining colocalized with Runx2 staining; **e-ii**, **f-ii** DAPI staining colocalized with Lpl staining; **a-iii**, **b-iii** DAPI staining colocalized with Runx2 and GFP staining; and **e-iii**, **f-iii** DAPI staining colocalized with Lpl and GFP staining. Blue indicates the nucleus shown by DAPI staining. Red indicates the nucleus shown by Runx2 staining in **a** and **b**, and the nucleus shown is by Lpl staining in e and f. PDL, periodontal ligament; NS, injection normal saline group; mBMMSCs, injection stem cell group. The scale bars in **a**, **b**, **e** and **f** are 50 μm, and the scale bars in **a-i**, **a-ii**, **a-iii**, **b-i**, **b-ii**, **b-iii**, **e-i**, **e-ii**, **e-iii**, **f-i**, **f-ii** and **f-iii** are 20 μm

To explore the fate of stem cells transplanted to the periodontal ligament, we performed double immunostaining of GFP with Runx2 and Lpl to determine cell fate after transplantation. mBMMSCs labelled with both Runx2-GFP and Lpl-GFP colonized the compression and tension sides of the orthodontic tooth (Fig. 4a, b, e, f). In the mBMMSC injection group, the number of Runx2-positive stem cells on the tension side was significantly greater than on the compression side (*P* < 0.05), but there was no significant difference in the number of Lpl-positive stem cells between the tension and compression sides (Fig. 4b, d, f, h). In the normal saline injection group, the number of Runx2-GFP- and Lpl-positive stem cells on the tension side was greater than on the compression side, but there were no significant differences. Furthermore, increased Runx2 expression was observed in the nucleus on the tension side (Fig. 4a, c, e, g).

### Effect of mBMMSCs on osteoclast differentiation in response to compression in vitro

**Fig. 5:**
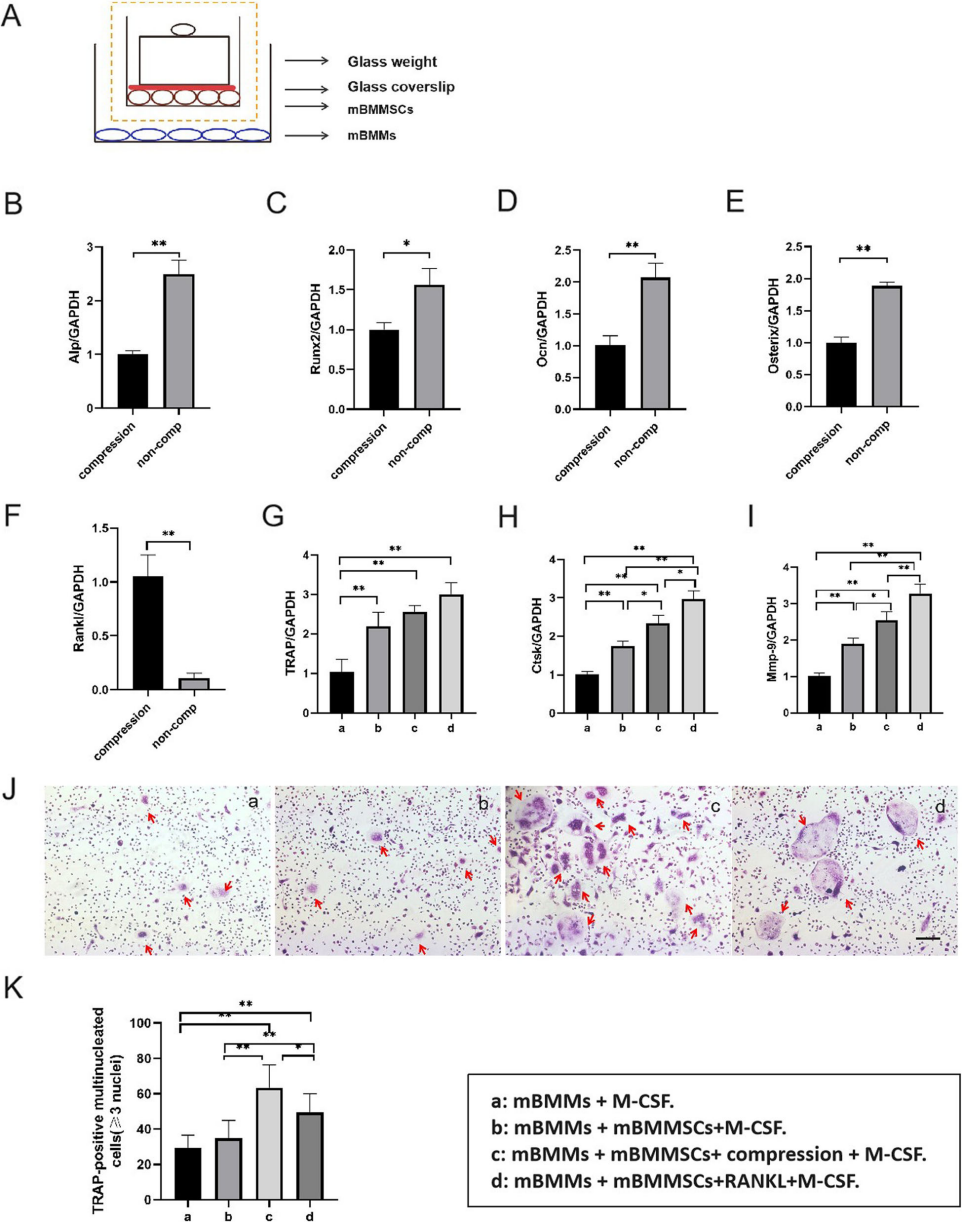
mBMMSCs under compression load promote osteoclast differentiation of mBMMs. **a** Schematic diagram of co-culture of mBMMs and stem cells under compression load. The orange dotted frame shows a schematic diagram of simply applying compressive force on the cell. **b**–**f** After simply applying compressive force on the mBMMSCs for 2 days, RT-PCR assay of Alp, Runx2, Ocn, Osterix and Rankl was performed. Results showed that expression levels of Alp, Runx2, Ocn and Osterix in the non-compression group were significantly higher than in the compression group, but Rankl expression in the compression group was significantly higher than in the non-compression group. *P < 0.05 versus compression group, **P < 0.01 versus compression group. **g**–**i** Gene expression of osteoclast differentiation markers in all four groups. When co-cultured with mBMMSCs under compression load, osteoclast differentiation markers (TRAP, Ctsk and Mmp-9) produced by mBMMs were approximately twice as high as those produced by mBMMs cultured independently. mBMMSCs under compression promoted expression of TRAP, Ctsk and Mmp-9 in mBMMs compared to mBMMSCs without compression. Gene expression of TRAP, Ctsk and Mmp-9 in mBMMs induced by Rankl and M-CSF was slightly higher than that induced by M-CSF in the co-culture of stem cells and mBMMs under compression. *P < 0.05, **P < 0.01. **j** TRAP staining images of mBMMs co-cultured with stem cells 7 days after compression in all four groups in vitro. The red arrows represent TRAP-positive multinucleated osteoclasts. The scale bars in **e** were 50 μm. **k** Semiquantitative analysis of the number of osteoclasts produced in the TRAP staining assay in all four groups in vitro. * *P* < 0.05, **P < 0.01

To verify the effect of mBMMSCs in response to compression on osteoclast differentiation, we performed in vitro experiments. First, to confirm the effect of mechanical load on the osteoclast differentiation of stem cells, we applied 2 g/cm^2^ mechanical force to mBMMSCs. A schematic diagram of the compressive force on the cell is shown in the orange dotted frame in Fig. 5a. After 2 days of culture, gene expression levels of Alp, Runx2, Ocn and Osterix in the non-compression group were higher than in the compression group (Fig. 5b–e, *P* < 0.01 for Alp, Ocn and Osterix, *P* < 0.05 for Runx2), while gene expression levels of Rankl in the compression group were higher than in the non-compression group (Fig. 5f, *P* < 0.01).

Next, we designed a cell co-culture system (Fig. 5a) and grouped it as shown below. After co-culture with stem cells under compression, gene expression of TRAP, Ctsk and Mmp-9 in mBMMs stimulated by M-CSF was higher than in mBMMs stimulated by M-CSF alone (*P* < 0.01 for TRAP, Ctsk and Mmp-9). Under M-CSF stimulation, gene expression of TRAP, Ctsk and Mmp-9 in mBMMs stimulated with Rankl was slightly higher than in mBMMs co-cultured with stem cells under compression (Fig. 5g–i). These results confirm that stem cells promote osteoclast differentiation in response to compression.

TRAP staining was used to detect the production of osteoclasts. Results showed that most osteoclasts were produced by M-CSF-induced mBMMs when co-cultured with stem cells under mechanical loading. The number was much higher than that produced by mBMMs stimulated by M-CSF alone and co-cultured with stem cells without mechanical load (*P* < 0.01) and slightly greater than that produced by mBMMs stimulated by Rankl and M-CSF (*P* < 0.05) (Fig. 5j, k).

## Discussion

BMMSCs, stem cells with multidirectional differentiation potential, have the ability to migrate to inflammatory and injury regions and have regulatory effects in these sites to promote local recovery and healing [22]. BMMSCs can regulate the activity of osteoblasts and osteoclasts through multiple secreted proteins, transcription factors, miRNAs and other unknown mechanisms. Recent evidence shows that BMMSCs exert their therapeutic ability through paracrine granules (extracellular vesicles), including exosomes, microbubbles and apoptotic bodies, which regulate the function of receptor cells by transmitting information carried by lipids, nucleic acids and proteins [23].

The significance of stem cells in the treatment of bone defects has been recently reported [24]. The combination of rat bone marrow stem cells and collagen is considered a promising method to repair alveolar bone defects after tooth extraction, preventing bone fenestration, bone dehiscence and periodontal risk during tooth movement [25]. Alveolar bone defects in patients with cleft lip and palate could be repaired using deciduous dental pulp stem cells, which produce satisfactory bone regeneration effects [26]. Therefore, stem cells play an important role in alveolar bone regeneration and metabolism. The main range of OTM is also in the alveolar bone, but the effect of stem cells on bone metabolism during OTM and its mechanism are not well characterized.

One previous study found that injection of GYY4137, a hydrogen sulphide (H_2_S) donor that enhances H_2_S levels and increases serum concentrations through systemic administration, accelerates tooth movement [27]. H_2_S, a gas transmitter produced from BMMSCs that regulates their osteogenic differentiation, is associated with bone homeostasis [28–30]. Mechanical loading of PDLSCs promotes expression of H_2_S in vitro and increases the number of TRAP-positive osteoclasts. Therefore, stem cells might promote tooth movement. To directly investigate the role of stem cells in tooth movement, for the first time, we injected homologous mBMMSCs into a mouse model of OTM through the tail vein. Ten days later, the distance of the first molar movement was measured by gross tooth tissue and micro-CT imaging. The distance of the first molar moving forward after injection of mBMMSCs was larger than in the control group. Histological observation revealed that the width of periodontal ligament on the compressed side of the tooth root was larger after injection of mBMMSCs compared to the control group, but there were no significant difference between the mBMMSCs group and saline regarding the width of the PDL. However, a trend towards increased PDL in the mBMMSCs group was observed, and the number of TRAP-positive osteoclasts in the pressure side was increased compared to the control. Therefore, in vivo injection of mBMMSCs increases differentiation of osteoclasts on the compressive stress side and increases the distance between teeth and alveolar bone, which facilitates tooth movement to the pressure side.

To track the location and function of stem cells after injection, we labelled the injected stem cells with GFP. After 3 days of tracing, green fluorescent staining showed that mBMMSCs were implanted in the periodontal ligament of orthodontic teeth, and most of them gathered on the compressed side, indicating that mechanical pressure was conducive to the recruitment of exogenous mBMMSCs. In previous studies, Kim found that PLA/β-TCP scaffolds containing kld12/kld12 substance P exhibited good recruitment ability for bone marrow mesenchymal stem cells after intravenous injection, indicating that the material promoted recruitment of stem cells into the skull defects of SD (Sprague Dawley) rats [31]. Zaky et al. [32] also found that in a rabbit model of ulnar defect implantation, PGS also promoted recruitment and differentiation of host osteoprogenitor cells in vivo, providing a good bone development environment. These studies indicate that osteogenic material implanted into the inflammatory site of bone defects increases recruitment of mesenchymal stem cells. In our study, stem cells were primarily recruited to the compression and osteoclastic sides rather than to the tension and osteogenic sides. It has been reported that the spleen undergoes inflammation induced by spinal cord injury after cervical spine crush injury. Umbilical cord mesenchymal stem cells collected in the spleen after tail vein injection. The authors believe that the spleen, a site of aseptic inflammation, can recruit stem cells [33]. In our study of OTM, the site of aseptic inflammation was on the compression side. Inflammation produces a variety of inflammatory factors to attract injected mBMMSCs. Exogenous mBMMSCs were first recruited to the compressed side of OTM, which may be due to the role of stem cells in inflammatory targets.

To understand the relationship between stem cells and the osteoclastic process of OTM, we conducted in vitro experiments to simulate compression of stem cells in vivo. In the past, some scholars collected the supernatant of periodontal ligament stem cells under pressure and cultured them with THP-1 human monocytes, which promote the induction of monocytes into macrophages [26]. In this study, we used a Transwell chamber to establish a new co-culture force system, which was more conducive to observing the induction of stem cells. The upper chamber used glass weights to exert pressure on stem cells, and osteoclastic differentiation of monocytes in the lower chamber was observed. Results showed that under the same stimulation of M-CSF, the number of osteoclasts in monocytes induced by mBMMSCs under pressure was much greater than that induced by mBMMSCs without pressure. Analysis of the gene expression of mBMMSCs after compression revealed that expression of Rankl was much higher than that of nonpressurized stem cells.

Osteoclast differentiation is primarily regulated by M-CSF and Rankl [31]. M-CSF mediates the survival and proliferation of early macrophage/osteoclast precursor cells, induces expression of nuclear factor receptor activator kappa B (RANK, receptor of RANKL), activates the RANKL/RANKL/OPG signalling pathway, and causes RANKL to combine with Rank on the membrane of osteoclast precursor cells [34]. This is a key pathway for osteoclast maturation [35]. When osteoclasts are activated, the internal structure of the cells changes. Actin, an important part of the cytoskeleton, rearranges and releases many cleavage enzymes, such as Mmp-9, TRAP, and Ctsk, that reach the absorption cavity through the folded boundary of osteoclasts, which is conducive to the migration of bone matrix and bone remodelling so that bone resorption continues [36, 37].

RANKL, a member of the tumour necrosis factor superfamily, plays an important role in osteoclastic differentiation [38]. It has been shown that RAW264.7 osteoclast precursor cells directly differentiate into osteoclasts in response to RANKL alone [39–42]. RANKL interacts with RANK to activate the downstream signalling molecule nuclear factor κB (NF-κB), which regulates expression of various osteoclast genes, such as MMP-9 and TRAP [43–47].

Therefore, in the experiment of osteoclast differentiation induced by co-culture in vitro, under the same stimulation of M-CSF, expression of Rankl in mBMMSCs with compressive stress was much higher compared to nonpressurized mBMMSCs, which combines with Rank, the receptor, produced by monocytes stimulated by M-CSF, inducing a large number of monocytes to differentiate into osteoclasts. The effect of inducing monocytes is even greater than that of adding RANKL protein directly. Therefore, osteoclast differentiation induced by stem cells after compression might not only increase expression of Rankl. Li reported that expression of growth differentiation factor 15 (GDF15) [14] was increased by pressure stimulation of human periodontal ligament cells, which played an important role in the activation of inflammatory factors and RANKL/OPG induced by pressure.

We also found that expression levels of TRAP, Ctsk and MMP-9 in stem cells under compressive stress and co-culture with monocytes were much higher than those in the non-compression stem cell and nonstem cell groups, indicating that the former exhibited more osteoclastic behaviour, and the results were basically consistent with the osteoclast count results. Similar to the findings of Chen’s co-culture experiment, PDLSCs promote the maturation of osteoclasts and promote expression of TRAP, CSTK and TRAF6 in RAW264.7 cells [48].

## Conclusions

In conclusion, compressive stress during OTM recruits stem cells, and the effect of compressive stress and Rankl on osteoclast differentiation induced by stem cells is synergistic, explaining our in vivo experimental results. Injection of stem cells into mice increases osteoclastic behaviour on the pressure side of orthodontic teeth and accelerates orthodontic movement. Stem cells regulate biological behaviour therapy. The effects of stem cells on osteoclast differentiation are complex and closely related to physical stimulation, autocrine or paracrine mechanisms, microenvironment stimulating factors and so on. We report the mechanism of osteoclastic regulation by stem cells in response to compression force during OTM and hope that this study highlights the clinical application of stem cells in the future.

## Data Availability

Data supporting the current study are available from the corresponding author upon reasonable request.

## Abbreviations

mBMMSCs: Mouse bone marrow mesenchymal stem cells
mBMMs: Mouse bone marrow-derived macrophages
OTM: Orthodontic tooth movement
PDL: Periodontal ligament
M-CSF: Macrophage colony-stimulating factor
MMP-9: Matrix metalloprotein-9
Ctsk: Cathepsin K
TRAP: Tartrate-resistant acid phosphate
Rankl: Nuclear factor kappa B receptor activating factor ligand
Runx2: Runt-related transcription factor
Alp: Alkaline phosphatase
Ocn: Osteocalcin
GFP: Green fluorescent protein
Lpl: Lipoprotein lipase
PDLSCs: Periodontal ligament stem cells
